# The influence of object-location binding mental load effects on the visual N1 and N2 Event-related Potentials

**DOI:** 10.1186/s13104-022-06086-0

**Published:** 2022-06-23

**Authors:** Solwoong Song, Jinsick Park, Young Min Park, In Young Kim, Dong Pyo Jang

**Affiliations:** Department of Biomedical Engineering, Hanyang University, Seoul, Korea

**Keywords:** EEG, ERP, Object-location binding memory, N2

## Abstract

**Objective:**

This study aimed to analyze the effect of object-location binding on the visual working memory workload. For this study, thirty healthy subjects were recruited, and they performed the “What was where” task, which was modified to evaluated object-location binding memory. We analyzed their ERP and behavior response.

**Results:**

Object memory and location memory were preserved during the task, but binding memory decreased significantly when more than four objects were presented. These results indicate that the N1 amplitude is related to the object-only load effect, and the posterior N2 amplitude is a binding-dependent ERP component.

## Introduction

VWM is essential for perception and information processing in the real visual world. VWM has a limited capacity determined by visual feature binding, not by the number of individual features, and is generally known to comprise three or four objects [1–3]. This binding memory function enables visual information processing even with a limitative VWM capacity. The effect of feature bindings on capacity has been investigated in several psychological studies. Feature bindings represent an object's identity-related features, such as color, shape, and orientation [4].However, the binding of object features and object location on VWM capacity has not been extensively studied. The VWM functional pathways can be divided into ventral stream features and dorsal stream features related to object identity and location [5–7]. Because object features and object location information are handled differently in the ventral and dorsal streams, the binding between the object features and the object location could be approached differently.

Most existing studies on object-location binding and VWM capacity have used change detection tasks [8–10]. They assessed object-location binding ability by how the ability to sense changes in whole-display or single-probe varied depending on how many objects were presented. However, it has been questioned whether the VWM load and capacity assessment using the change detection task is accurate [11]. To prevent location information from being encoded in an abstract configuration, such as a relative inter-object position, spatial reconstruction allows each object's actual coordinates to be memorized directly is better [9, 12]. Pertzov reported a new task to directly address a memorized object's identity and location that should reveal object-location binding ability more effectively [13, 14]. In Pertzov’s “What was where” task, participants are asked to identify an object in a memory array between a foil object and one of the presented objects and localize its original location. They calculated the memory accuracy for both the identification-object memory and the localization-location memory and also calculated the object-location binding memory accuracy when both identification and localization were accurate.

It is well known that the ventral stream and the dorsal stream converge in the medial temporal lobe [15]. Lesion or functional brain-imaging studies have shown that the hippocampus is the most important region for object-location binding [16–18]. However, another study reported that VWM capacity is intact after damage to the medial temporal lobe structures [19].

In the EEG studies, there are several well-known ERP components correlated with visual information processing. The visual N1 is elicited from the primary visual cortex in the early stage of visual information processing and is posteriorly distributed at 150–200 ms [20–24]. It represents the orienting of attention [20], discrimination mechanisms [21], and object-based selection [22]. Also, there is a posterior N2 (also known as the N2c), and it is one of the negative subcomponents with a near 200-ms latency [25]. The posterior N2 is known to reflect the stimulus classification subprocess and is observed in visual attention paradigms but has not been studied as much as other N2 subcomponents [25–28].

Based on these results, we assumed that object-location binding load and capacity might be correlated with an earlier occipital stage of visual information processing. Thus, this study aimed to analyze the effect of object-location binding on the VWM workload by analyzing behavioral data and ERP response in the occipital region while performing an object-location binding memory task.

## Main text

### Material and methods

#### Participants

The protocol for this study was approved by Hanyang University Institutional Review Board (HYI-17-048-4). Thirty people (15 males, 15 females) aged 19–28 years (mean age: 21.73 ± 2.44 years) were recruited voluntarily. All of the participants were university students who had no neurological problems.

### Experimental paradigm

**Fig. 1:**
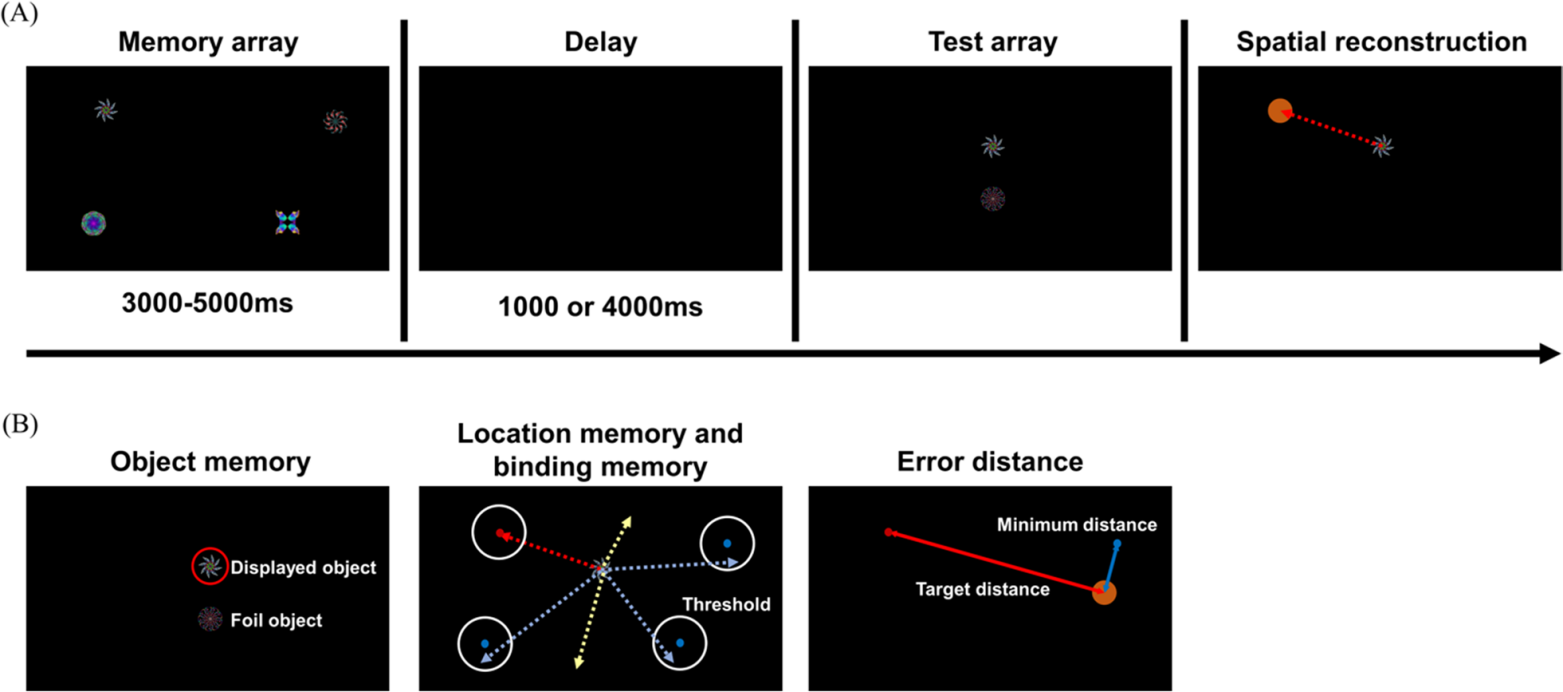
**A** Examples of a single trial of task. Trial consists of memory array, delay, test array (identification), and spatial reconstruction (localization). **B** Measurements of behavioral performance; Object memory, location memory and error distance

For this study, the "What was where" task was modified to observe the mental load effect, similar to our previous study [29]. Participants performed a modified Pertzov’s paradigm to evaluate object-location binding memory. The task was presented using MATLAB (Natick, Massachusetts: The MathWorks Inc.). In this task, a few fractal objects (130 × 130 pixels) were displayed in random locations on a 53.2 × 29.9 cm (1920 × 1080 pixels) touchscreen monitor for 3–5 s (Fig. 1A). The length of time that objects were presented on the screen increased in proportion to the number of objects. We showed two objects after a slight delay: the displayed fractal object and a foil fractal object. Then, participants had to select one object and drag it to the remembered location using the touchscreen. The total task consisted of three sessions, and each session had 150 trials. Three, four, or five objects were presented in each session. The order of the session was chosen at random. This change in the number of presented objects was carried out to observe the mental load's effect during encoding. Response coordinates were recorded to calculate the error distance and memory accuracy. The correct localization thresholds for location memory and binding memory were determined using a visual angle of 4.5° [13, 14, 29]. The corresponding approximate threshold in pixels was 125 pixels, and it was calculated by considering the viewing distance of 42 cm and the size of the viewing monitor (Fig. 1B).

### Measurement and analysis

To assess each participant's performance, the object memory accuracy was measured using the percentage of correctly identified fractals in the test arrays (Fig. 1B). The accuracy of location memory was determined by the percentage of correctly identified locations of selected fractals. The binding memory accuracy was defined as the number of trials in which participants located a target fractal object in its original location. Swap error ratio was defined as ratio of trials in which participants located a target fractal object in other objects’ location. We analyzed the behavioral performance according to the number of objects in the behavioral data. ANOVA and independent t-test for each pair were performed using with the Statistical Package for the Social Sciences (SPSS) Statistics Release 24 software (IBM, Somers, NY, USA).

The EEG was recorded during the task using a gel type 64-channel 10–20 system EEG cap with a BRAINBOX EEG-1166 amplifier system (Braintronics, Almere, FL, NL). EEG data were sampled at 1024 Hz, and impedances for all electrodes was kept below 10 kΩ. We used active G1/G2 ground reference (G1: FPz, G2: AFz) of BRAINBOX system for ground and reference. Common averaging reference and bandpass filtering (1–30 Hz) were performed. Eye movements and blinks were removed using independent component analysis (ICA) [30]. Then we segmented EEG data of the trial in which both the identity and the location of the object were correctly answered into encoding-lock epochs from -200 ms to 800 ms. Baseline removal and amplitude normalization were performed for each epoch. Contaminated epochs were rejected automatically with FASTER [31] which is fully automated statistical thresholding for the EEG artifact rejection algorithm. These data were used to calculate the encoding-lock ERPs.

## Results and discussion

### Behavioral performance

**Fig. 2:**
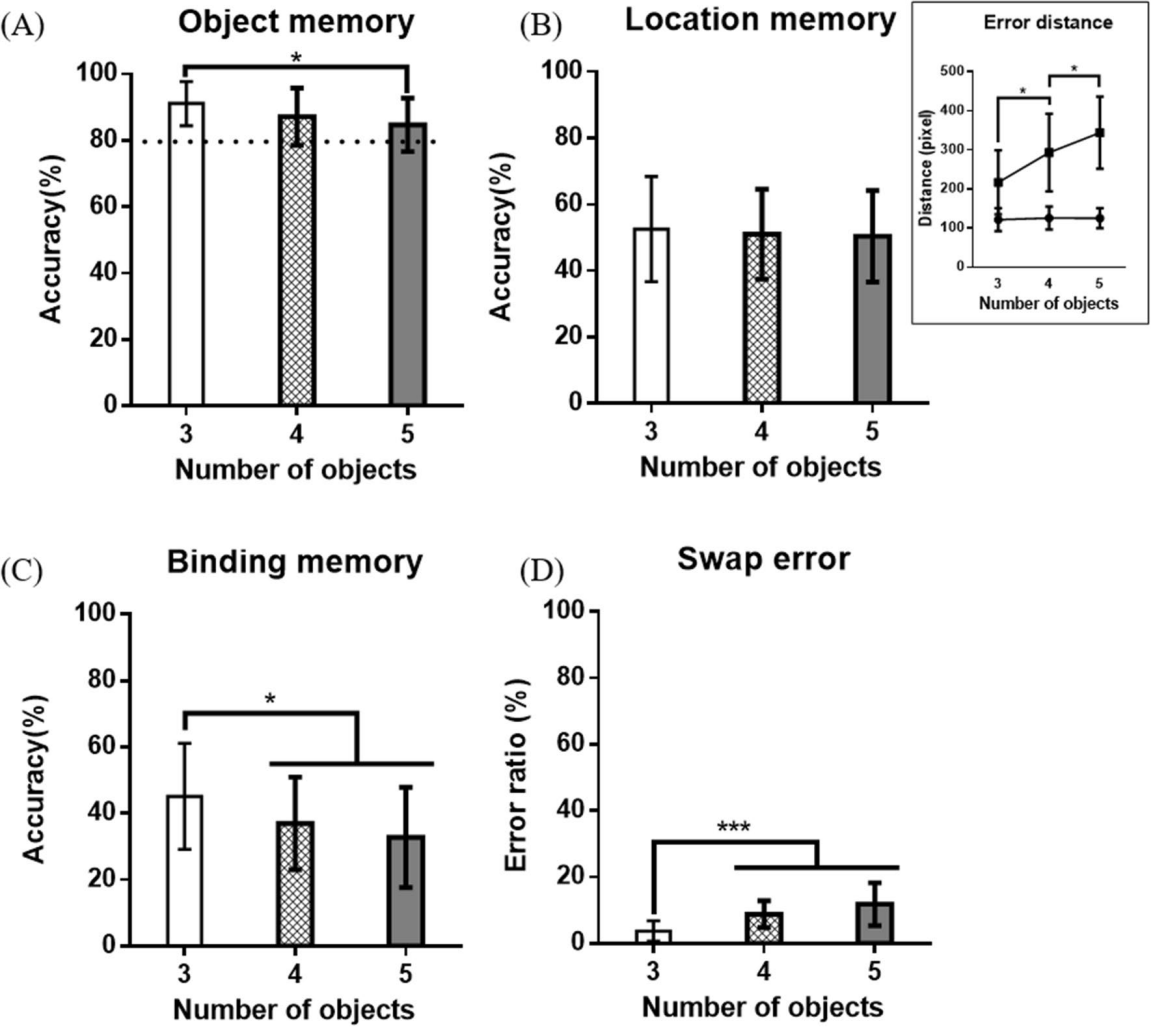
The mean value and memory accuracy for (**A**) object memory, (**B**) location memory and error distance, (**C**) object-location binding memory and (**D**) swap error ratio (*p < 0.05, ***p < 0.0005)

As shown in Fig. 2A, in object memory, statistically, significant differences were shown only when three objects were presented and when five objects were presented (p = 0.0014). In location memory, no statistically significant change was observed even when the number of objects increased. In the case of binding memory and swap error ratio, there were statistically significant differences when three objects were presented, and four/five objects were presented (Binding memory: p = 0.0391/p = 0.0032, Swap error ratio: p < 0.0005/ p < 0.0005), but there was no difference between four and five object presentation. As the number of presented objects increases, the error distance between the target and actual response coordinates tended to increase. However, the error distance from the nearest neighborhood did not increase even if the number of objects increased as shown in the Fig. 2C

### ERP

**Fig. 3:**
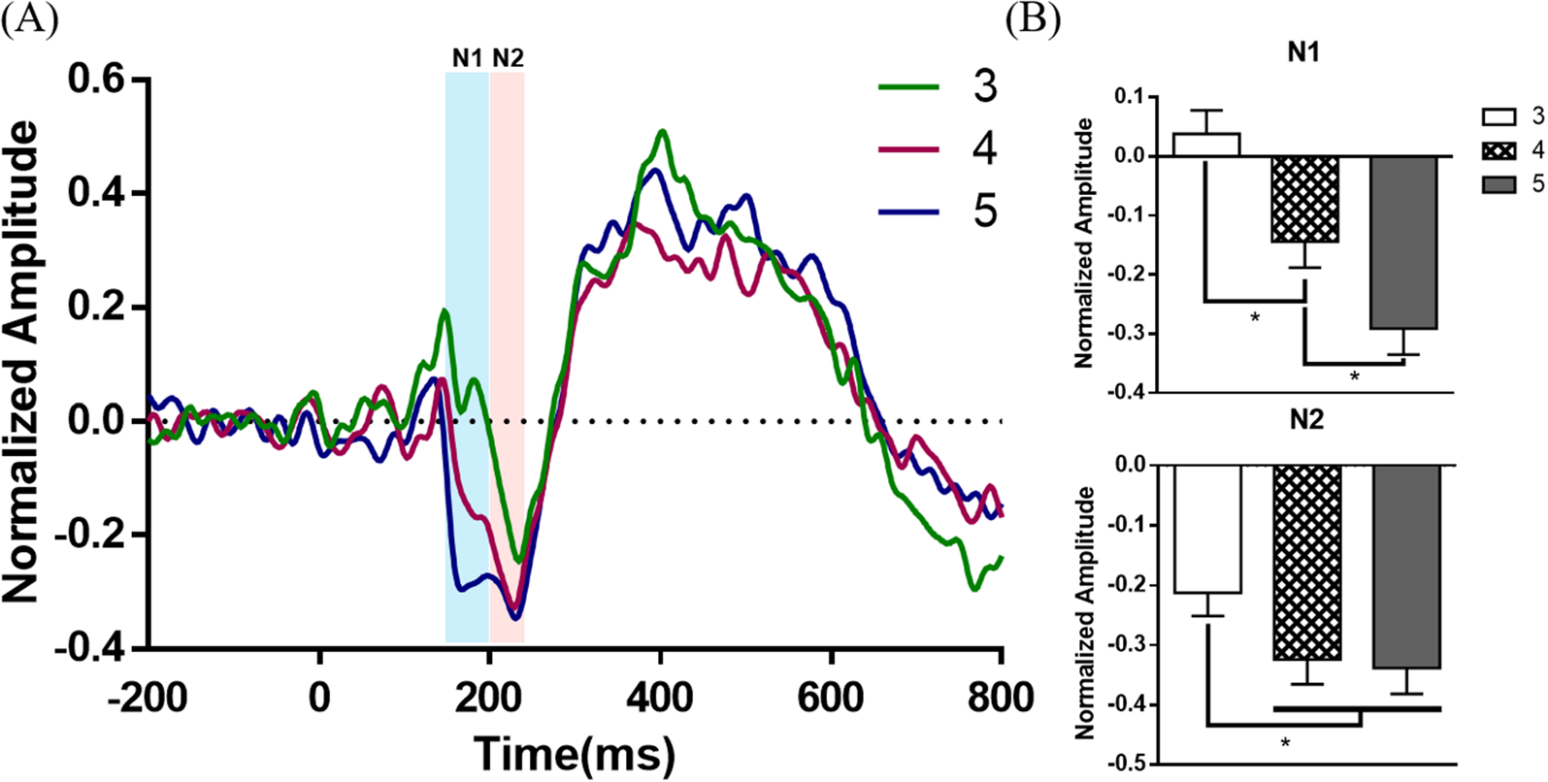
**A** Encoding-lock ERPs on posterior electrode site (POz). The ERPs' values were calculated and illustrated separately for each presented object (green line: 3 objects, red line: 4 objects, blue line: 5 objects). **B** The mean value and standard error of the mean of the ERP components’ normalized amplitude on POz

We identified two distinct ERP components on the posterior electrode sites (Fig. 3). Two consecutive negative peaks at N1 (150–200 ms) and N2 (200–240 ms) appeared at parieto-occipital electrode sites. We visually checked single-trial ERPs and confirmed that these were not the same peak with a different latency. The amplitudes of N1 tended to increase when the number of presented objects increased from 3 to 4 (p < 0.0001) and from 4 to 5 (p = 0.0478). The amplitude of N2 increased as the number of presented objects increased from 3 to 4 (p = 0.0286) and from 3 to 5 (p = 0.03). In contrast to N1, when the number of presented objects increased from 4 to 5, a significant change was not observed (p = 0.9325).

## Discussion

In the object memory accuracy, there was a significant difference between when three objects were presented and when five objects were presented. However, regardless of the number of objects presented, the object memory accuracy was more than 90 percent under all conditions, which means that only the objects’ identity was memorized, but the VWM capacity limit was not reached even though the object presented in this paradigm was complex.

Location memory accuracy was somewhat less accurate for all set sizes. It can be interpreted that there was a partial loss of location memory in all cases, but the error distance shows that the minimum distance did not increase even if the number of presented objects increased to five. This means that the location memory accuracy was evaluated much lower than actual, because of we set very tight localization threshold to detect binding memory and swap error, and in fact location memory was kept almost constant with no difference in all cases.

Binding memory accuracy and swap error ratio are related to object-location binding memory, and both of their values were low because of the tight localization threshold, which was similar to that of localization memory. They showed a significant difference between 3 and 4 but not between 4 and 5. This pattern is similar to the commonly known VWM capacity limit [2, 4]. Our findings for object memory, location memory, and binding memory indicated that only binding memory ERP components elicited from the presented objects provided more information.

Two consecutive ERP components, N1 and N2 was elicited on parieto-occipital site by the paradigm. The amplitude of N1 increased in proportion to the number of presented objects. It can be said that there was a load effect with an increasing number of presented objects in N1. This result agrees with the consecutive results of studies that have reported perceptual load effects on the N1 [32]. As noted for the behavioral results' object memory accuracy, a capacity limit at the N1 load effect was not found. Based on the similarity of these trends and previous research on visual N1, we think that N1 is related to the object-only load effect. In N2, the amplitude increased when the number of presented objects increased from 3 to 4, but not when it increased from 4 to 5. This change can be interpreted in terms of object-location binding memory accuracy variability with an increase in the number of objects presented from 3 to 4, but not when the number was increased from 4 to 5. This pattern was different from the pattern in which the N1 and object memory accuracy changed with an increasing number of presented objects. Therefore, we assumed that the N2 is an object-location binding dependent component, and its amplitude might reflect the task difficulty of the binding process. And also this N2 related process occurs in the parieto-occipital cite, as early as 200 ms after seeing the objects.

The amplitude of N2 was saturated when four objects were presented and there were no significant changes between four and five objects, as same as binding memory accuracy and swap error ratio. However, the amplitude of N1 and object memory accuracy did not show the same change. This seems to have occurred because the object-only difficulty was easy despite the load effect.

In conclusion, the N1 amplitude is related to the object-only load effect, and the posterior N2 amplitude is a binding-dependent ERP component.

## Limitations

In this study, there is only ERP analysis about electroencephalogram without source localization analysis like Low Resolution Electromagnetic Tomography. Source localization analysis might help to figure out each step of object-location binding memory process.

## Data Availability

The datasets used and analysed during the current study are available from the corresponding author on reasonable request.

## Abbreviations

VWM: Visual working memory
EEG: Electroencephalography
ERP: Event-related potentials
ICA: Independent component analysis
FASTER: Fully automated statistical thresholding for EEG artifact rejection algorithm
